# P-581. Warming Climates and Emerging Threats: The Influence of Climate Variability on Vector-Borne Disease Transmission in the United States

**DOI:** 10.1093/ofid/ofaf695.795

**Published:** 2026-01-11

**Authors:** Maria Akiki, Ali Hemade, Pascal Salameh

**Affiliations:** University of Connecticut, Hartford, CT; Lebanese University, Beirut, Beyrouth, Lebanon; Lebanese University, Beirut, Beyrouth, Lebanon

## Abstract

**Background:**

Climate change is altering the epidemiology of vector-borne diseases by influencing vector survival, transmission dynamics, and geographic spread. Dengue, malaria, and West Nile virus (WNV) are key mosquito-borne illnesses in the U.S., with incidence shaped by rising temperatures, shifting precipitation patterns, and global travel. This study examines long-term national trends using a retrospective ecological time-series approach.

**Figure:**
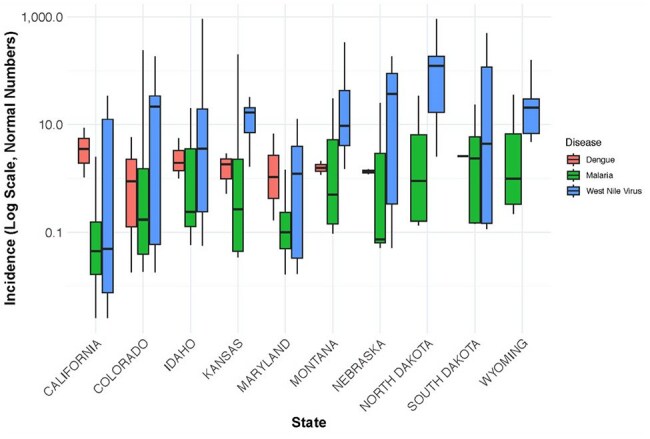
Distribution of Vector-Borne Disease Incidence in the Top 10 U.S. States (Log Scale) Boxplot showing the distribution of dengue (red), malaria (green), and West Nile virus (blue) incidence across the top 10 U.S. states with the highest cumulative vector-borne disease burden from 1980 to 2023. Incidence is displayed on a log scale (per 100,000 population) to highlight variability and outliers. West Nile virus shows the widest range and highest incidence, particularly in Great Plains states such as South Dakota, North Dakota, and Nebraska.

**Figure:**
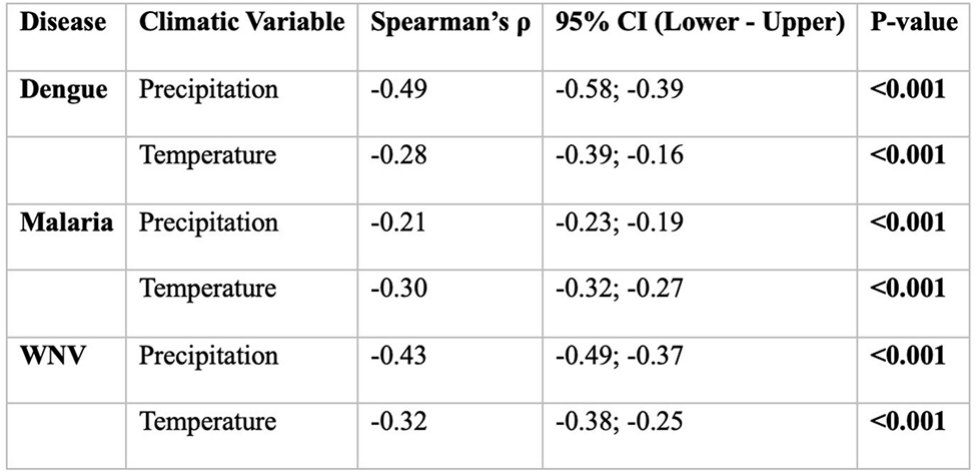
Spearman Correlation Between Climate Variables and Vector-Borne Disease Incidence Spearman’s ρ coefficients assessing the strength and direction of association between monthly temperature and precipitation and the incidence of dengue, malaria, and West Nile virus in the U.S. from 1980 to 2023. All correlations were statistically significant (p < 0.001). Negative correlations suggest that lower precipitation and temperature were associated with higher disease incidence across all three infections.

**Methods:**

This retrospective time-series study examined the association between climate and the incidence of dengue, malaria, and WNV in the U.S. from 1980 to 2023. Disease data and climate data were obtained from Project Tycho, and the Parameter-elevation Regressions on Independent Slopes Model, respectively. We used correlation analyses, generalized additive models, and ARIMAX forecasting to assess climate-disease relationships.

**Results:**

WNV had the highest and most variable incidence, concentrated in drier states such as South Dakota, Nebraska, and Colorado. Dengue showed strong seasonality with peaks in late summer and early fall, primarily in southern states. Malaria cases were mainly imported and showed a stable but low seasonal trend, with sporadic cases reported primarily in states with high international travel volume, including California, New York, and Florida (Figure 1).

Spearman analysis of climate associations demonstrated that WNV incidence had a negative correlation with precipitation (p < 0.001). Dengue incidence correlated positively with elevated temperatures and humidity (p = 0.008). Malaria incidence exhibited a weaker but statistically significant association with climate variables (Figure 2). GAMs revealed significant nonlinear effects of precipitation on WNV and malaria (p < 0.001), but not dengue. ARIMAX models projected increased WNV and dengue incidence under continued warming and dry conditions.

**Conclusion:**

Climate variability strongly influences vector-borne disease trends in the U.S. WNV thrives in dry, high-risk areas, dengue is expanding in the warm, humid south, and malaria remains primarily travel-related but climate-sensitive. Our projections showed rising WNV and dengue incidence with continued warming, highlighting the need for climate-informed public health interventions.

**Disclosures:**

All Authors: No reported disclosures

